# Redox-driven cardioprotective effects of sodium-glucose co-transporter-2 inhibitors: comparative review

**DOI:** 10.1186/s12933-023-01822-7

**Published:** 2023-04-29

**Authors:** Julia Hoehlschen, Dominik Hofreither, Tamara Tomin, Ruth Birner-Gruenberger

**Affiliations:** 1 Institute of Chemical Technologies and Analytics, TU Wien, Wien, Austria; 2 Diagnostic and Research Institute of Pathology, Medical University of Graz, Graz, Austria

**Keywords:** SGLT-2, Empagliflozin, Canagliflozin, Dapagliflozin, Heart failure, Oxidative stress

## Abstract

Sodium-glucose co-transporter-2 inhibitors are used in the treatment of diabetes but are also emerging as cardioprotective agents in heart diseases even in the absence of type 2 diabetes. In this paper, upon providing a short overview of common pathophysiological features of diabetes, we review the clinically reported cardio- and nephroprotective potential of sodium-glucose co-transporter-2 inhibitors currently available on the market, including Dapagliflozin, Canagliflozin, and Empagliflozin. To that end, we summarize findings of clinical trials that have initially drawn attention to the drugs’ organ-protective potential, before providing an overview of their proposed mechanism of action. Since we particularly expect that their antioxidative properties will broaden the application of gliflozins from therapeutic to preventive care, special emphasis was put on this aspect.

## Introduction

In 2021, 537 million people worldwide (10.5% of the global population) lived with diabetes and another 541 million adults were at high risk to develop diabetes due to impaired glucose tolerance [1]. More than 90% of diabetic patients suffer from type 2 diabetes, manifested with pancreatic β-cell dysfunction and insulin resistance [1, 2]. Subsequent insulin deficiency and accompanying hyperglycemia adversely affect diverse micro- and macrovascular processes, including diabetic kidney and cardiovascular diseases.

**Fig. 1:**
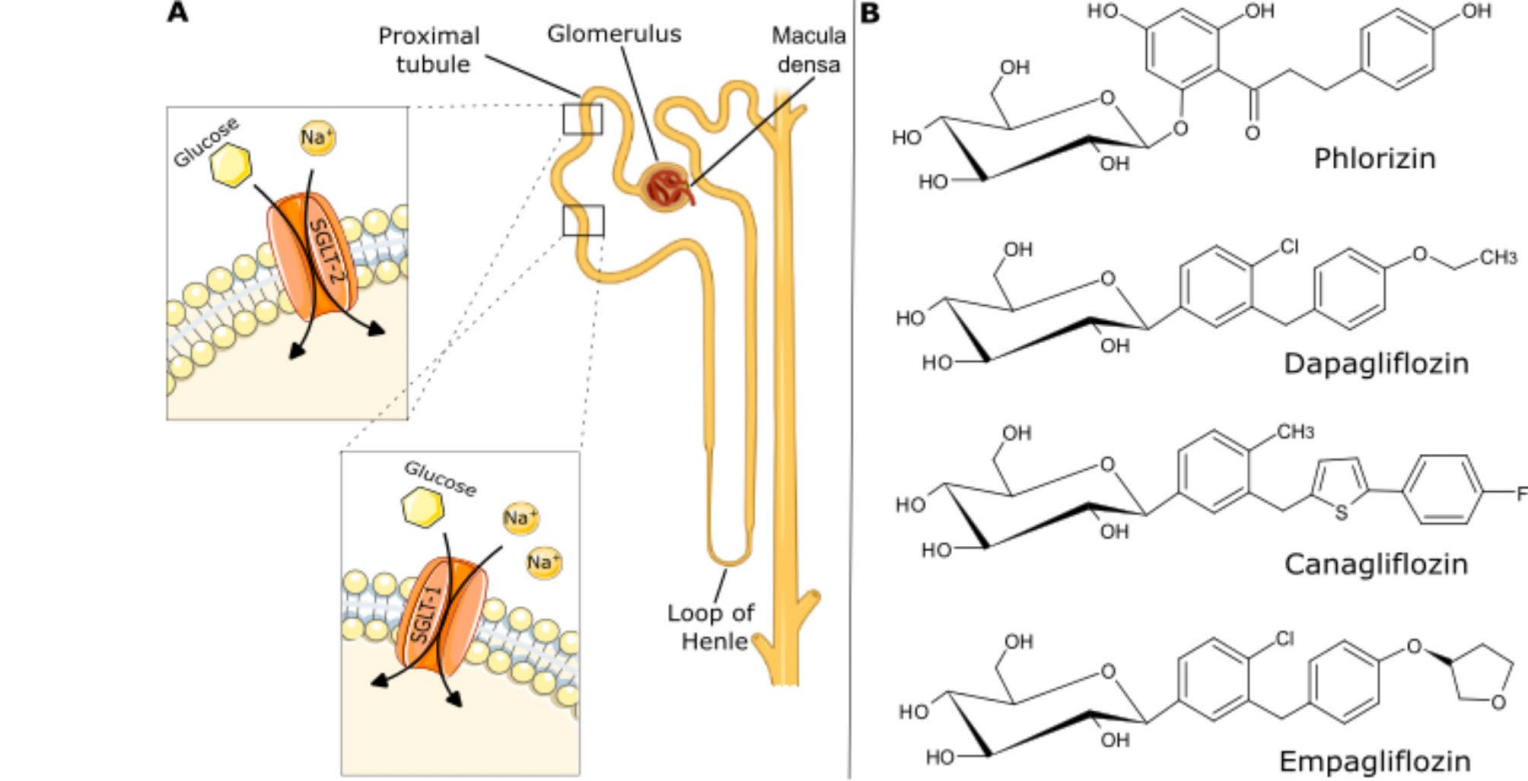
SGLT-2 expression and inhibitor structure. (A) Localization of SGLT-1 and 2 within the proximal tubule. (B) Chemical structures of Dapa-, Cana- and Empagliflozin in the order of their approval and their precursor Phlorizin.

Reduction of blood glucose has been shown to improve microvascular complications such as nephron- and neuropathy, but to be insufficient to prevent the death from cardiovascular events, the most relevant macrovascular complication of type 2 diabetes [2]. Therefore, new drug therapies offering cardioprotective properties are of great interest. One such emerging drug class are sodium-glucose co-transporter-2 (SGLT-2) inhibitors, a relatively novel anti-diabetic medication. Under conditions of functional SGLT and normoglycemia, SGLT-1 and 2 are responsible for about 3% and 97% of renal glucose reabsorption, respectively [3]. The stoichiometry of glucose transport via SGLT is one Na^+^ ion per glucose molecule for the high-capacity/low-affinity transporter SGLT-2 and two Na^+^ ions per glucose molecule, for the low-capacity/high-affinity transporter SGLT-1 [4, 5]. While SGLT-2 was so far known to be exclusively expressed in the S1 and S2 segments of the early proximal tubule’s luminal membrane (Fig. 1A), SGLT-1 is mainly located in the S3 segment and the intestine’s brush border membrane but was found in heart, liver and lung as well [6, 7]. In diabetes, the renal transport maximum for glucose can be increased by inducing tubular growth or raising the expression of SGLT-1 and 2, contributing to the state of hyperglycemia [3, 8]. Correspondingly, inhibition of SGLT, especially of SGLT-2, leads to glucose secretion via the urine (glycosuria) [9]. In 2012, the first SGLT-2 inhibitor Dapagliflozin (Fig. 1B) derived from its precursor Phlorizin (a non-specific SGLT inhibitor, Fig. 1B [8]) was approved by the European Medicines Agency (EMA) for the treatment of type 2 diabetes. Dapagliflozin was later followed by Canagliflozin and Empagliflozin (Fig. 1B) among others. Over the past years it has become increasingly recognized that SGLT-2 inhibitors exhibit tissue-protective effects beyond their systematic, glucose-lowering properties. Here (regardless of the patient’s diabetic status) kidney and heart are the organs benefitting the most. In this review, we summarize the clinically reported organ-protective potential of SGLT-2 inhibitors currently available on the market, before specifically focusing on their cardioprotective, antioxidative properties, a trait that will most likely propel this drug class from curative to preventive care.

## Renal complications related to diabetes and proposed protective mechanisms of gliflozins

According to a retrospective study by Ling et al. (2020), vascular complications in damaged organs account for 26.8% of all end-stage diabetic related deaths, being one of its leading causes. Of this percentage, 71.1% of death cases resulted from kidney failure, making it the most affected organ [10]. Attempting to cope with hyperglycemia, diabetic patients struggle with renal hyperfiltration (manifested by an increased glomerular filtration rate (GFR)) which results from high glomerular pressure and increased sodium and glucose reabsorption via SGLT-1 and 2. Here, the diabetes-driven boost in Na^+^ reabsorption in the proximal tubule, causes a decreased Na^+^ delivery to the macula densa leading to a reduced tubuloglomerular feedback (TGF), accompanied by increasing filtration rates [11]. When inhibiting this process by SGLT-2 inhibitors, sodium delivery to the macula densa is enabled and hyperfiltration rates are reduced [12]. Upon SGLT-2 inhibition, the highly increased sodium cannot be sufficiently cleared by the Na^+^/K^+^ ATPases of macula cells, resulting in an osmotic gradient leading to macula cell swelling and leakage of adenosine triphosphate (ATP) from the basolateral membrane, which is extracellularly converted to adenosine. Triggered adenosine signaling leads to Ca^2+^ mediated vasoconstriction, reducing blood flow and, in consequence, glomerular pressure [13, 14]. On a metabolic level, SGLT-2 inhibitors are proposed to act as stabilizers of hypoxia-inducible factor 1 alpha (HIF-1a), diverting cellular pathways from oxidative metabolism to glycolysis [13, 15]. Hypoxia represents a critical, often detrimental point in end-stage renal diseases but, though counterintuitive, strengthened HIF-1a and 2a signaling has been shown to improve the expression of oxygen-sensitive, reno-protective genes which can aid cell recovery under ischemic conditions [16, 17]. Studies in diabetic mice as well as isolated kidney cells have demonstrated the ability of Empagliflozin to reprogram renal metabolism and to improve mitochondrial function and antioxidative defense [18, 19]. Lastly, glycosuria is recognized as a signal of nutrient-deficiency — especially in kidneys — leading to the activation of autophagy. This in turn rescues the cells from damaged mitochondria and reduces overall oxidative stress burden [20]. For more detailed information on renal effects of gliflozins we direct the reader to other excellent reviews [13, 17, 20, 21].

## Cardiovascular complications related to diabetes: focus on oxidative stress

Cardiovascular events such as heart failure (HF) are multicausal, with metabolic syndrome, insulin resistance and diabetes mellitus representing major risk factors [22]. The main pathophysiology of HF is the decreased efficiency of the cardiac muscle to pump blood through the circulatory system [22]. To preserve homeostasis and contractile function in response to stress, the heart offers compensation strategies, including cell growth as well as increase in angiogenesis, energy efficiency, autophagy, and antioxidant generation [23, 24]. Though essential, these adaptive processes might result in fibrosis, altered sarcomere structure, impaired Ca^2+^ handling, induction of fetal gene programming, mitochondrial dysfunction, disbalance in reactive oxygen species (ROS), metabolic remodeling and cell death, potentially leading to progressive HF in the long term [23–27].

The heart is constantly consuming a variety of energy substrates to fuel life-long contractions. Energy metabolism is governed by multiple layers of crosstalk among metabolic pathways and cellular energy state, as well as substrate and oxygen availability [24, 28]. While glycolysis is the most important route for glucose after cellular uptake, it (and glucose metabolism overall) only contributes little to the total ATP generation in the heart. Oxidative phosphorylation (OXPHOS), utilizing the generated redox equivalents from the metabolization of various energy substrates, is responsible for 95% of ATP production in healthy cardiomyocytes [24, 29, 30]. Thus, high mitochondrial activity and an extensive number of oxidative organelles require non-enzymatic (e.g. glutathione) and enzymatic (e.g., superoxide dismutases, catalase, peroxiredoxins, thioredoxin and glutathione peroxidase systems) antioxidative attenuation of the occurring electron leakage and generated ROS, such as peroxides or free radicals [31, 32]. Under physiological conditions, ROS assert essential signaling functions for the regulation of mitochondrial activity and cellular adaptions to stressors. NADPH oxidases such as NOX4 represent a major source of ROS in cardiac cells, participating in the modulation of the immune response and mitochondrial biogenesis following exercise [31, 33]. However, prolonged exposure to oxidative stress followed by ROS accumulation leads to impaired antioxidative defense, dysregulation of redox signaling and damage to macromolecules, consequently advancing the functional decline of the myocardium [31, 34–36].

**Fig. 2:**
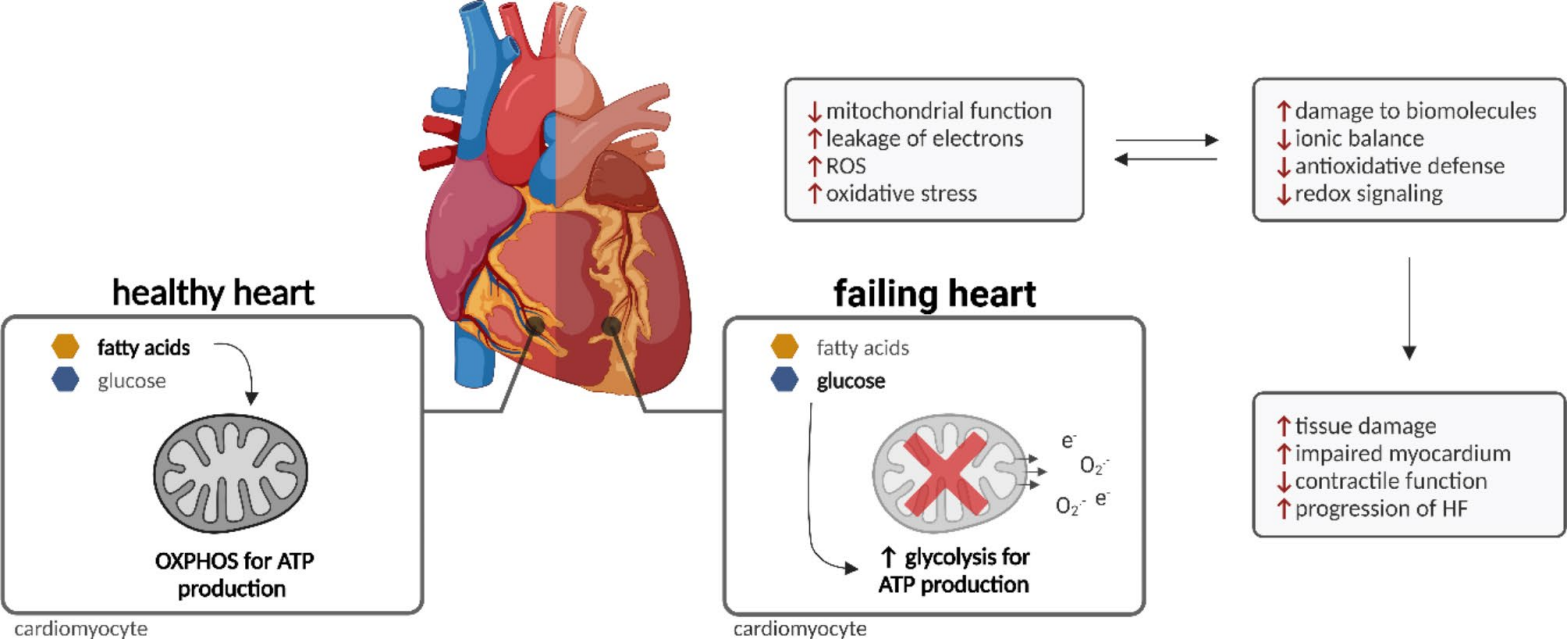
The progression of heart disease can be associated with profound metabolic remodeling, mitochondrial dysfunction and disturbed redox homeostasis. These interconnected changes may lead to energy depletion, impaired cardiac function and ultimately heart failure.

Heart diseases in various stages can be linked to disrupted and abnormal metabolism in cardiac cells. While healthy cardiomyocytes primarily rely on fatty acids (FA) for ATP production, with beta-oxidation and mitochondrial activity tightly synchronized, impaired cardiomyocytes during pathological remodeling may ultimately show reduced fatty acid oxidation and rely on glucose as fuel instead, as shown in Fig. 2 [24, 37]. This is considered a compensatory mechanism associated with pressure overload and energy depletion, as indicated by elevated levels of intracellular adenosine monophosphate (AMP) in the early pathogenesis of HF [24, 30, 38]. Indeed, failure to increase glucose transport and glycolysis in this AMP-activated protein kinase (AMPK) cascade-dependent way has been shown to advance disease progression in transgenic animal models [39–42]. The pleiotropic cardioprotective effects of AMPK activation have been suggested not to be fully exclusive to energy repletion [43–48]. However, prolonged activation of the glycolytic phenotype may result in decompensation and HF progression. The adaptive increase in glycolysis may not be matched in glucose oxidation and lead to an uncoupling between substrate uptake and oxidation, further impairing cellular metabolic function and redox homeostasis [24, 26, 49, 50].

In the failing heart, oxidative stress is not only linked to dysregulated cardiac metabolism but also to systemic factors such as diabetes, metabolic syndrome and ageing [34, 35]. Hyperglycemia, dyslipidemia, reduced metabolic flexibility and constant low-grade inflammation have been associated not only with increased ROS but also with excess carbonyl stress and the formation of advanced glycation and lipoxidation end-products [51]. Additional alterations of the insulin-resistant heart may result in FA-induced lipotoxicity and further uncoupling of OXPHOS. Namely, disturbance of the mitochondrial membrane structure and Ca^2+^ homeostasis, as well as increased leak respiration, have all been reported in diabetic heart disease [26, 50, 52]. Restoring metabolic capability, mitochondrial function, and, ultimately, energy output in (diabetic) HF may be essential to ameliorate its progression and facilitate treatment [53, 54].

## Cardioprotective effects of SGLT-2 inhibitors from clinical trials

In 2015, the first long-term study on gliflozins, including 7,028 patients, was published. The aim of the EMPA-REG Outcome™ trial [55] was to determine long-term effects of Empagliflozin on cardiovascular safety in type 2 diabetes patients (≥ 18 years) with a high risk of cardiovascular events as well as its cardioprotective effects. Patients suffering from type 2 diabetes, having a glycated hemoglobin value (HbA_1c_) in a certain range and established cardiovascular diseases were randomly divided into three groups (1:1:1) receiving Empagliflozin (10 mg or 25 mg) or placebo once daily. The background glucose-lowering therapies were kept unchanged during the first 12 weeks but were allowed to be adjusted afterwards [55]. The results showed significant differences between the Empagliflozin and placebo groups regarding lower risk of death from cardiovascular causes, death from any causes and hospitalization due to heart failure. However, there were no significant differences evident between the doses of Empagliflozin (10 mg vs. 25 mg) [56]. Glycated hemoglobin levels were lowered in patients of the Empagliflozin groups after 12 weeks with decreasing impact after adjustment of background therapies and over time. It was further found that drug intake led to slight reduction in weight, waist circumference, uric acid level and blood pressure, systolic as well as diastolic. Levels of LDL and HDL cholesterol were slightly increased [56].

Similar results in terms of positive cardiovascular effects were obtained in a long-term study on Canagliflozin (CANVAS) in which 10,142 patients suffering from type 2 diabetes were included and randomized to receive Canagliflozin (100 mg or 300 mg) or placebo (1:1:1) [57]. However, in CANVAS, patients were either ≥ 30 years and had a history of symptomatic atherosclerotic cardiovascular disease or ≥ 50 years with at least two risk factors for cardiovascular diseases, for instance high systolic blood pressure, a history of diabetes (≥ 10 years), or being a current smoker [57]. One noticeable difference in the adverse side effects (compared to the Empagliflozin trial) was a higher risk of amputations of toe or metatarsal and increased bone fractures. It has to be emphasized though that the highest absolute risk correlated with an already existing history of amputation or peripheral vascular disease in respective patients [57].

A long-term study on Dapagliflozin (DECLARE–TIMI 58) revealed a reduced occurrence of cardiovascular death, myocardial infarction or ischemic stroke in the Dapagliflozin group compared to the placebo. However, contrary to the previous trials these findings were not significant [58]. 17,160 patients were included, suffering from type 2 diabetes, who were ≥ 40 years of age and had an established atherosclerotic cardiovascular disease (40.6%) or multiple risk factors for it (59.4%) [58] The inclusion criteria of all three trials are presented in Table 1.

**Table 1:** Overview of the most important inclusion criteria in SGLT-2 trials on type 2 diabetes patients as well as their outcomes, incl. hazard ratios and confidence intervals (CI). In EMPA-REG patients received Empagliflozin, for CANVAS patients were treated with Canagliflozin and DECLARE-TIMI 58 was carried out to study the effect of Dapagliflozin.

	EMPA-REG	CANVAS	DECLARE-TIMI 58
No. of patients	7,028	10,142	17,160
Inclusion criteria			
	Type 2 diabetes	Type 2 diabetes	Type 2 diabetes
	HbA_1c_ ≥ 7% and ≤ 10%		
	≥ 18 years	≥ 30 years or ≥ 50 years (s.b.)	≥ 40 years
*Cardiovascular (CV) diseases*	Established CV diseases	a) ≥ 30 years and history of symptomatic CV diseases **or** b) ≥ 50 years and ≥ 2 risk factors for CV diseases (duration of type 2 diabetes ≥ 10 years, systolic blood pressure > 140 mmHg (blood pressure–lowering treatment), current daily cigarette smoker, documented micro- or macroalbuminuria, or high-density lipoprotein (HDL) cholesterol of < 1 mmol/l (< 39 mg/dl)	a) Est. CV (40.6%) **or** b) ≥ 2 risk factors for it (age (≥ 55 years male ≥ 60 years female), dyslipidemia, hypertension, current tobacco use) (59.5%)
*Medication*	Possible, incl. insulin	Possible, antihyperglycemic agents as mono- or combination therapy	Possible with antihyperglycemic agents
Outcomes			
*Cardiovascular*	Significant lower risk of death from CV (hazard ratio, 0.62 (95% CI, 0.49 to 0.77)) or any cause (hazard ratio, 0.68 (95% CI, 0.57 to 0.82))	Reduced occurrence of death from CV causes, nonfatal myocardial infarction, nonfatal stroke (hazard ratio, 0.86 (95% CI, 0.75 to 0.97))	Reduced occurrence of CV death, myocardial infarction or ischemic stroke (hazard ratio, 0.93 (95% CI, 0.84 to 1.03))
*Hospitalization*	Significant lower risk of hospitalization for heart failure (hazard ratio, 0.65 (95% CI, 0.50 to 0.85))	Lower risk of hospitalization for heart failure (hazard ratio, 0.67 (95% CI, 0.52 to 0.87))	Lower risk for hospitalization for heart failure (hazard ratio, 0.83 (95% CI, 0.73 to 0.95))
*Other*	Lower glycated hemoglobin levels (after 12 weeks with decreasing impact), slight reduction in weight, waist circumference, uric acid level and blood pressure (systolic and diastolic); slight increase of LDL and HDL cholesterol	Lower glycated hemoglobin levels, slight reduction in weight, waist circumference, blood pressure (systolic and diastolic); slight increase of LDL and HDL cholesterol	Lower glycated hemoglobin levels, slight reduction in weight and blood pressure (systolic and diastolic)
*Adverse side effects*	Genital infections	Genital infections, higher risk of amputations (toe or metatarsal) and increased bone fractures with existing burden	Genital infections

More recently, in 2019 and 2020, two studies were published, investigating whether the positive cardiovascular effects of Dapagliflozin and Empagliflozin would be restricted to type 2 diabetes patients. The DAPA-HF trial included 4,744 patients (≥ 18 years) suffering from chronic heart failure with a reduced heart function of which 42% had a known diagnosis of type 2 diabetes, 3% were newly diagnosed and 55% did not have a background of diabetes. During the study, all patients received standard heart-failure device therapy as well as standard drugs [59]. Inclusion criteria for the EMPEROR-Reduced (Empagliflozin outcome trial in patients with chronic heart failure with reduced ejection fraction) trial was very similar to DAPA-HF (see Table 2) and included 3,730 participants aged ≥ 18 years, one half each diagnosed or not diagnosed with diabetes, respectively [60]. In both studies a reduced incidence for hospitalization due to HF or death from cardiovascular causes was observed upon treatment with both SGLT-2 inhibitors regardless of diabetic conditions [59, 60]. Subsequently, approvals of both drugs have been extended to an application in heart diseases even in the absence of type 2 diabetes by the EMA [61]. Moreover, renal protective effect of Empa, Dapa- and Canagliflozin was attested in the respective trials and is being further investigated (61).

**Table 2:** Overview of the most important inclusion criteria in DAPA-HF and EMPEROR Reduced trials, including patients suffering from heart diseases, independent on diabetes state. Further, the outcomes of the trials are summarized, incl. hazard ratios and confidence intervals (CI).

	DAPA-HF	EMPEROR-Reduced
**No. of patients**	4,744	3,730
**Inclusion criteria**		
	≥ 18 years	≥ 18 years
	Ejection fraction ≤ 40% and New York Heart Association (NYHA) class II, III, IV symptoms	Left ventricular ejection fraction ≤ 40% and New York Heart Association (NYHA) class II, III, IV symptoms
*Medication*	Required; Angiotensin-converting-enzyme inhibitor, angiotensin-receptor blocker, sacubitril-valsartan plus beta-blocker, mineralocorticoid receptor antagonist	Required incl. Diuretics, inhibitors of the renin-angiotensin system and neprilysin, beta-blockers, mineralocorticoid receptors antagonists
**Outcomes**	Reduced incidence of hospitalization due to HF (hazard ratio, 0.70 (95% CI, 0.59 to 0.83) or death from CV (hazard ratio, 0.82 (95% CI, 0.69 to 0.98), observation of renal protective effect	Reduced incidence of hospitalization due to HF (hazard ratio, 0.69 (95% CI, 0.59 to 0.81) or death from CV (hazard ratio, 0.92 (95% CI, 0.75 to 1.12), observation of renal protective effect

## Potential cardioprotective mechanisms of SGLT-2 inhibitors beyond systemic effects

The trials’ results emphasize that the cardioprotective capability of SGLT inhibitors cannot be solely explained by their glucose-lowering effect [9, 62]. Several experiments have been carried out aiming to provide insights into the underlying key mechanisms.

Treatment of cardiomyocytes from healthy rats and rabbits cultivated in medium containing 5 mM or 10 mM glucose supplemented with Empagliflozin (1 µM) reduced cytoplasmic concentrations of Na^+^ and Ca^2+^ and increased mitochondrial Ca^2+^ concentrations, reversing the negative effects of increased glucose concentrations [63]. Furthermore, Empagliflozin showed properties similar to those of an inhibitor of the cardiac Na^+^/H^+^ exchanger (NHE) that seemed to be independent of SGLT-2 expression and glucose [63]. Treatment of these animal cells with Empagliflozin followed by subsequently NHE inhibitor Cariporide only minimally affected the change in cytoplasmic Na^+^ concentration chosen as indicator for NHE inhibition [63]. The same was true for treatments in reverse order (Cariporide immediately followed by Empagliflozin) [63]. Measuring the alteration of cytoplasmic Na^+^ concentration and the activity of NHE in healthy mouse cardiomyocytes in the presence of Empa-, Dapa- and Canagliflozin, dosed similarly to the plasma concentrations reached by respective therapies in humans (1 µM, 1 µM and 3 µM, respectively), a decrease in cytosolic concentration of Na^+^ was found in all three conditions [64]. Furthermore, *in silico* docking studies on the extracellular Na^+^ binding site of NHE proved high binding affinity for all three inhibitors (-34.3 kJ/mol Empagliflozin, -32.2 kJ/mol Dapagliflozin, -37.2 kJ/mol Canagliflozin) compared to negative control glucose (-24.3 kJ/mol) [64]. Their glucoside moieties were oriented towards the Na^+^ binding site, the aglycone part lining the extracellular opening of that site [64]. However, Chung et al. have contradicted the direct inhibition of NHE1 by Empa-, Cana- and Dapagliflozin [65]. The inhibitors’ effects were compared with Cariporide in conditions of intracellular acidosis to evoke large NHE1 fluxes and best show inhibitory effects. While Cariporide showed an inhibited H^+^ flux in cardiomyocytes, this flux was not affected by the SGLT-2 inhibitors [65].

On metabolic level, the three drugs, Cana-, Empa- and Dapagliflozin, seem to exhibit differential effects. In contrast to Canagliflozin, which significantly activated AMPK in HEK293 cells and murine hepatocytes already at concentrations similar to those in human plasma when treated with the drug, Empa- and Dapagliflozin showed weaker effects even at higher therapeutic doses [66]. Increasing concentrations of Canagliflozin led to an increase in ADP:ATP ratios, suggesting reduced cellular oxygen consumption through inhibition of complex I of the respiratory chain [66]. Similar findings were made in human umbilical vein endothelial cells (HUVEC) and human vascular smooth muscle cells (HaoVSMC) [67]. A study on prediabetic male rats (absent hyperglycaemia), suffering from chronic vascular complications and renal function impairment, has shown that, among other findings, non-esterified fatty acid levels were increased upon Empagliflozin treatment, assumingly leading to the generation of ketone bodies [68]. Beyond that, a conversion from glucose to fatty acid oxidation as energy source was described [68]. Moreover, anti-inflammatory properties of Canagliflozin were observed that might be explained by AMPK activation [67]. AMPK activation accompanied by inhibited mTORC1 activity was also found upon treatment of diabetic and non-diabetic mice with Dapagliflozin, interpreted by the authors as possible explanation for its organ-protective effect [69]. Another effect of this activation was shown to be the inhibition of NFκB signaling in functional studies as well as the involvement in the inhibition of IL-6-stimulated Janus kinase-STAT signaling by directly leading to phosphorylation of Janus kinase-1 in human endothelial cells [70].

## SGLT-2 inhibitors and oxidative stress

**Fig. 3:**
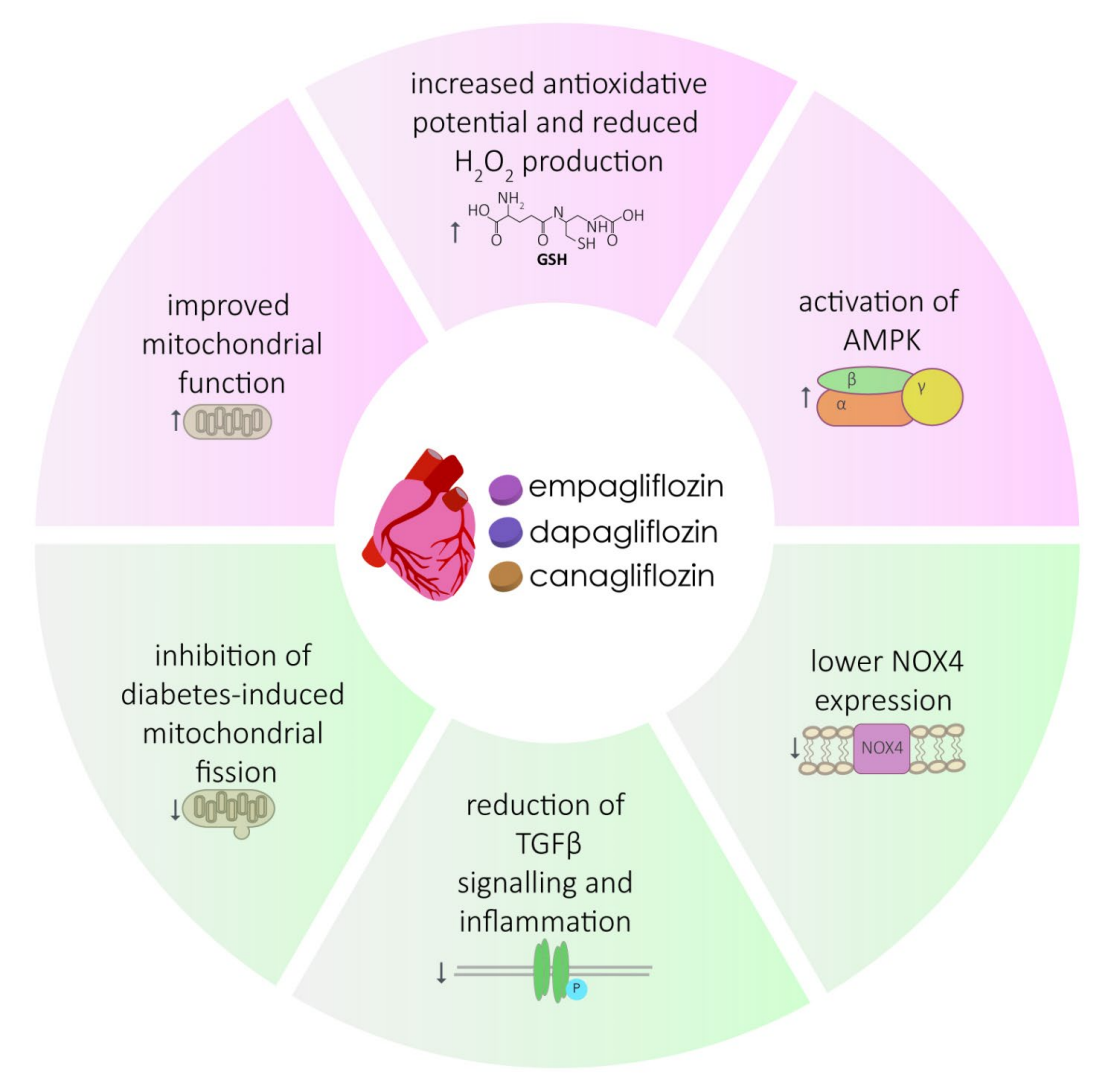
Overview of SGLT2 inhibitors and their proposed mechanisms of oxidative stress relieving effects, including improvement in mitochondrial function, better electron coupling, activation of AMPK and antioxidant production as well as reduction of pro-inflammatory signaling.

The first report on the ability of SGLT2 inhibitors to reduce oxidative stress traces back to the initial studies of their effects on hyperglycemia in type 2 diabetes [71]. Experiments in monocytes and endothelial cell lines revealed that the cell’s disfunction in hyperglycemic conditions could be improved by Empagliflozin in a glucose transport-independent mechanism, leading to an attenuated ROS accumulation [72]. The effect of Empa-, Cana- and Dapagliflozin on protease activating receptor 2 (PAR2), whose actions (the widening of blood vessels) are suggested to be compromised due to oxidative stress, were tested [71] in experiments on mouse aorta-derived endothelial cells grown under hyperglycemic (25 mM glucose) and normoglycemic (10 mM glucose) conditions [71]. Treatment with any of the three SGLT-2 inhibitors protected the endothelium from the increased glucose uptake and consequent augmented mitochondrial ROS production, sustaining function of the endothelium [71]. Empagliflozin was found to exert a two-phase effect depending on its concentration (≤ 1 µM: protection of vascular endothelial function; 5 to ≥ 10 µM: no vasodilation or smooth muscle contractile responses anymore) and to improve mitochondrial function, as indicated by a reduced proton leak measured via oxygen consumption rates [71]. Furthermore, treatment with Empagliflozin significantly decreased the amount of H_2_O_2_ in mitochondria and cytosol, alleviated oxidative parameters, such as 3-nitrotyrosine, glutathione (GSH), lipid peroxide, and reversed the pathological repression of the protein kinase G (NO-sGC-cGMP-PKG) pathway [73]. eNOS-dependent PKGIα oxidation and polymerization were significantly reduced by Empagliflozin and led to its translocation back to the cytosol [73]. Markers of oxidative stress (lipid hydroperoxide, glutathione peroxidase (GSH-Px), superoxide dismutase (SOD) and malondialdehyde (MDA)) were also investigated in tissue from diabetic mouse hearts treated and not treated with Empagliflozin. Their levels were found to be significantly higher (lipid hydroperoxide, MDA) or lower (SOD, GSH-Px) in diabetic untreated mice compared to Empagliflozin treated ones [74]. The same was observed in the atrial muscle of diabetic and non-diabetic rats [75]. Besides this, the expression of NOX4 — the highest abundant NAD(P)H oxidase isoform in cardiomyocytes — was highly increased in untreated diabetic mice [74]. Among others, the level of the transcription factor Nrf2 (NF-E2-related factor 2), that binds to the antioxidant responsive element (ARE), was markedly increased in the treated mouse heart tissue, hinting that oxidative stress levels were reduced [74]. Furthermore, fibrosis was suppressed by Empagliflozin treatment through inhibition of the transforming growth factor β (TGF-β)/SMAD pathway due to downregulation of TGF-β expression by Empagliflozin and/or elevated levels of Smad7, a negative inhibitor of the aforementioned pathway [74]. The positive effect of Empagliflozin on microvascular morphology incl. fibrosis and membrane thickening was further confirmed by treating diabetic cardiac microvascular endothelial cells (CMEC) with Empagliflozin. The high levels of mitochondrial and intracellular ROS produced by the cells could both be restricted by this treatment [76]. In addition, diabetes induced mitochondrial fission was also prevented by Empagliflozin, which brought the ratio of AMP:ATP closer to the one observed in control cells, leading to the activation of AMPK [76]. Similar findings were also reported for reperfusion injury, where Empagliflozin was able to preserve mitochondrial function through AMPK activation [77]. Markers of microvascular inflammation (ICAM-1, CVAM-1, TNF-α, IL-6) which are markedly increased in heart failure with preserved ejection fraction (HFpEF) were found to be significantly reduced after treatment of fibers from healthy and HFpEF human hearts with Empagliflozin [73] and Canagliflozin [67]. A study in non-diabetic failing pig hearts also showed that Empagliflozin positively influenced cardiac metabolism typically found in failing hearts by diverting ATP production to beta oxidation and ketogenesis, decreasing the consumption of carbohydrates and thereby approaching the conditions found in healthy hearts [9]. Proposed protective mechanisms of SGLT2 inhibitors are also summarized in Fig. 3.

## Conclusion

SGLT-2 inhibitors show excellent promise for cardio-preventive care and, as demonstrated, cell culture experiments provide a good starting point in understanding how SGLT-2 inhibitors are involved in mechanisms improving cardiovascular conditions. However, most of the studies focused on hyperglycemic conditions [66, 73, 74, 76, 78] and not all were carried out in cardiomyocytes [66, 67, 76, 78]. Some of the clinical studies also searched for protein biomarkers in the blood which could tell us more regarding the underlying effects of the drugs. In case of the EMPEROR study (Empagliflozin), comparing samples taken right before the treatment’s start (baseline), after 12 weeks and 52 weeks of treatment, showed no significant differences in protein expression between the two timepoints during the treatment, hinting that the effect of Empagliflozin is time-independent. However, a small group of intracellular proteins involved in autophagic flux, weakening of oxidative stress and inflammation processes in the heart as well as promotion of repair and regeneration in heart and kidney were found to be differentially expressed upon treatment with Empagliflozin [79]. This finding potentially explains the often-described positive effects of gliflozins on these two organs. The highest increase in protein abundance was found for insulin-like growth factor-binding protein (IGFBP1), transferrin receptor protein 1 (Tfr1) and erythropoietin (EPO), which is functionally related to hemoglobin increase, presumably one of the major benefits of SGLT2 inhibitors for heart and renal function [79].

Overall, both* in vitro* and *in vivo* studies agree in one thing: SGLT-2 inhibitors are able to protect our heart independent of their glucose lowering effects. However, more research is ahead of us before we are able to comprehensively decipher their direct and indirect cellular targets and corresponding mechanism of action, in order to maximize their therapeutic potential.

## Data Availability

Not applicable.

## List of abbreviations

AMP: Adenosine monophosphate
AMPK: AMP-kinase
ARE: Antioxidant responsive element
ATP: Adenosine triphosphate
CMEC: Cardiac microvascular endothelial cells
CV: Cardiovascular
EMA: European Medicines Agency
EPO: Erythropoietin
FA: Fatty acids
GFR: Glomerular filtration rate
GSH(-Px): Glutathione (peroxidase)
HaoVSMC: Human vascular smooth muscle cells
HbA_1c_: Glycated hemoglobin value
HDL: High-density lipoprotein
HF: Heart failure
HFpEF: Heart failure with preserved ejection fraction
HIF-1a: Hypoxia-inducible factor 1 alpha
HUVEC: Human umbilical vein endothelial cells
IGFBP1: Insulin-like growth factor-binding protein
LDL: Low density lipoprotein
MDA: Malondialdehyde
NADPH: Nicotinamide adenine dinucleotide phosphate
NHE: Na^+^/H^+^ exchanger
Nrf2: NF-E2-related factor 2
NYHA: New York Heart Association
OXPHOS: Oxidative phosphorylation
PAR2: Protease activating receptor 2
PKG: Protein kinase G
ROS: Reactive oxygen species
SGLT: Sodium-glucose co-transporter
SOD: Superoxide dismutase
Tfr1: Transferrin receptor protein 1
TGF: Tubuloglomerular feedback
TGF-β: Transforming growth factor β
